# Sub-ppb level HCN photoacoustic sensor employing dual-tube resonator enhanced clamp-type tuning fork and U-net neural network noise filter

**DOI:** 10.1016/j.pacs.2024.100629

**Published:** 2024-06-28

**Authors:** Lihao Wang, Haohua Lv, Yaohong Zhao, Chenglong Wang, Huijian Luo, Haoyang Lin, Jiabao Xie, Wenguo Zhu, Yongchun Zhong, Bin Liu, Jianhui Yu, Huadan Zheng

**Affiliations:** a Key Laboratory of Optoelectronic Information and Sensing Technologies of Guangdong Higher Education Institutes, and Department of Optoelectronic Engineering, Jinan University, Guangzhou 510632, China; b Guangdong Key Laboratory of Electric Power Equipment Reliability, Electric Power Research Institute of Guangdong Power Grid Co., Ltd., Guangzhou, Guangdong 510080, China; c Guangdong-Hong Kong-Macao Joint Laboratory for Intelligent Micro-Nano Optoelectronic Technology, School of Physics and Optoelectronic Engineering, Foshan University, Foshan 528225, China

**Keywords:** Photoacoustic spectroscopy, Quartz tuning fork, Trace gas sensor, Quartz-enhanced photoacoustic spectroscopy

## Abstract

Hydrogen cyanide (HCN) is a toxic industrial chemical, necessitating low-level detection capabilities for safety and environmental monitoring. This study introduces a novel approach for detecting hydrogen cyanide (HCN) using a clamp-type custom quartz tuning fork (QTF) integrated with a dual-tube acoustic micro-resonator (AmR) for enhanced photoacoustic gas sensing. The design and optimization of the AmR geometry were guided by theoretical simulation and experimental validation, resulting in a robust on-beam QEPAS (Quartz-Enhanced Photoacoustic Spectroscopy) configuration. To boost the QEPAS sensitivity, an Erbium-Doped Fiber Amplifier (EDFA) was incorporated, amplifying the laser power by approximately 286 times. Additionally, a transformer-based U-shaped neural network, a machine learning filter, was employed to refine the photoacoustic signal and reduce background noise effectively. This combination yielded a significantly low detection limit for HCN at 0.89 parts per billion (ppb) with a rapid response time of 1 second, marking a substantial advancement in optical gas sensing technologies. Key modifications to the QTF and innovative use of AmR lengths were validated under various experimental conditions, affirming the system's capabilities for real-time, high-sensitivity environmental monitoring and industrial safety applications. This work not only demonstrates significant enhancements in QEPAS but also highlights the potential for further technological advancements in portable gas detection systems.

## 1 Introduction

In recent years, significant strides have been achieved in the theory and design of artificial neural networks, particularly in signal processing. It is imperative to cultivate a thorough comprehension of fundamental neural network structures and their profound implications on signal processing algorithms and applications [1]. With the progression of computer technology, neural networks have witnessed escalating utilization in estimation tasks. In comparison to conventional state estimation methods like the Wiener filter and Kalman filter, neural networks offer a distinct advantage in handling uncertainty factors and demonstrate applicability in highly nonlinear systems [2].

Hydrogen cyanide (HCN) is a molecule of substantial industrial and environmental importance. This compound is utilized in a variety of industrial applications, from synthesizing adiponitrile for nylon production to formulating specialized pharmaceuticals [3]. However, the utility of HCN is overshadowed by its significant toxicity, which has resulted in its classification as a chemical warfare agent [3], [4]. The National Institute for Occupational Safety and Health (NIOSH) has highlighted the dangers associated with HCN exposure, setting a stringent short-term exposure limit at 4.7 ppmv, emphasizing the risks posed by even low concentrations of this compound [5], [6], [7].

In the context of space missions, HCN detection becomes critically significant. In the closed environment of a spacecraft, potential hazards such as fire, system malfunctions, or unintended chemical releases demand immediate monitoring [8], [9]. Primary fire risks stem from solid materials, including overheated wires, cable bundles, and circuit boards [10], the combustion of which releases various trace gases including HCN [11]. Recognizing this, the National Aeronautics and Space Administration (NASA) has prioritized the development of fire detection technologies for manned spacecraft cabins, targeting the detection of HCN as early indicators of potential fires [12]. In their pursuit, NASA is advancing gas sensing technology to enhance the safety and emergency response of manned spacecraft in case of a fire [4], [5], [6], [9]. Additionally, HCN has been identified in planetary atmospheres during exploration missions [13]. On the earth, the primary anthropogenic sources of HCN include vehicle emissions and metallurgical processes [3]. The need for HCN sensors is thus underscored by their widespread industrial use, environmental impact, and relevance in specialized areas such as space exploration. The advancement of HCN detection methods is a crucial juncture between enhancing safety and scientific advancement.

In recent years, there has been significant progress in HCN gas detection technology, enhancing both sensitivity and response time [14]. Photoacoustic spectroscopy (PAS) has become a favored method for trace gas detection due to its robustness and compactness [15], [16]. PAS operates on the principle that modulated light sources excite molecules from their ground states to excited states [17], [18]. The subsequent de-excitation by vibrational transduction (V-T) relaxation produces periodic heating, generating acoustic waves [19], [20], [21]. To avoid 1/*f* noise and ambient background disturbances, modulation frequencies greater than 1 kHz are typically employed [22], [23]. The amplitude of the acoustic signal *S* in PAS can be expressed by (1), (2) [24], [25]:

(1) S∼αQP/ƒ

(2) α=σN

where *α* is the absorption coefficient, *Q* is the quality factor of the resonator, *P* is the effective optical power, *f* is the modulation frequency, *σ* is the absorption cross-section, and *N* is the concentration of gas molecules, respectively. A unique advantage of PAS is its independence from the light absorption path length [26], [27]. Signal enhancement can be effectively achieved by increasing the laser excitation power [28], [29]. PAS distinguishes itself from other spectroscopic methods by not requiring an optical detector; instead, an acoustic transducer is used to convert acoustic waves into electrical signals [30], [31]. These attributes afford PAS with high sensitivity within a compact and robust sensor design [32], [33], [34], with applications spanning medical diagnostics, chemical analysis, industrial processes, and environmental monitoring [25], [32], [35].

Quartz-enhanced photoacoustic spectroscopy (QEPAS), a derivative of PAS, substitutes the conventional microphone-based approach with a quartz tuning fork (QTF) [36], [37], [38]. QTFs are piezoelectric elements of millimeter-scale, routinely integrated into clocks and electronic circuits to maintain time [39], [40], [41], [42]. The QTFs exhibit a high *Q*-factor of approximately 10,000 and a narrow resonant frequency bandwidth of only a few Hz at standard atmospheric pressure, even with metal casings removed [43], [44], [45], [46]. This sharp resonance enhances the selectivity of QEPAS and its immunity to ambient noise [47], [48], [49], [50]. The QEPAS spectrophone, comprising a QTF and an acoustic micro-resonator (AmR), leverages the AmR, typically made of stainless-steel capillaries, to foster acoustic resonance and amplify the signal-to-noise ratio (SNR) [51]. Numerous QEPAS spectrophone variations have emerged over the past decades, aiming to elevate QEPAS sensor performance [52], [53], [54], [55]. Initially, QEPAS employed commercially available watch QTFs since 2002. However, to improve optical collimation and leverage molecular V-T relaxation, Spagnolo et al. introduced custom tuning forks in 2014 [56]. These custom forks, featuring novel vibration modes [57], [58], octupole electrodes [59], and optimized T-shaped prongs [60], [61], marked a significant enhancement. Most recently, Zheng et al. have reported a novel clamp-type QTF with an aperture of approximately 1 mm and a *Q*-factor exceeding 10,000 [51]. This clamped QTF, built upon the cost-effective and resonantly comparable watch QTF, differs from previous custom designs.

In this work, we unveil the application of a clamp-type custom quartz tuning fork (QTF) coupled with a dual-tube acoustic micro-resonator (AmR) aimed at highly sensitive HCN photoacoustic gas sensing. A pair of specifically designed longitudinal AmRs were introduced to work in conjunction with the clamp-type custom QTF. The AmR geometry was meticulously optimized through theoretical calculations and empirical validation, resulting in a robustly coupled on-beam QEPAS configuration. The on-beam configuration, where the laser beam passes directly through the gap between the prongs of the quartz tuning fork (QTF) and is aligned with the acoustic micro-resonator (AmR), was chosen for its superior optical alignment and energy coupling efficiency. This setup facilitates stronger photoacoustic coupling and improved signal strength compared to the off-beam configuration, making it ideal for achieving high sensitivity and low detection limits in HCN detection. Recognizing the direct proportionality of QEPAS sensitivity to the optical excitation power, the inclusion of an Erbium-Doped Fiber Amplifier (EDFA) proved substantially advantageous [62], [63], [64]. The EDFA was utilized to amplify the laser power by approximately 286-fold. The increased laser power enhances the photoacoustic signal due to the greater number of absorbed photons, which generate stronger acoustic waves, thereby improving the signal-to-noise ratio (SNR). This leads to more accurate and reliable detection of trace gas concentrations. However, while the EDFA amplifies the laser power, it can also cause a slight degradation in beam quality. To mitigate the potential increase in noise due to this degradation, we optimized the structure of the quartz tuning fork, effectively maintaining a high SNR and ensuring a substantial improvement in sensitivity. This optimization allows the system to detect lower concentrations of hydrogen cyanide (HCN) by amplifying the acoustic response and achieving a lower detection limit. To improve the sensor performance, we employed a neural network filtering method based on machine learning, known as the transformer-based U-shaped neural network, tailored to refine the photoacoustic signal and effectively mitigate background noise. This multifaceted approach culminated in attaining a detection limit for HCN as low as 0.89 parts per billion (ppb). With an expedient response time of 1 second for online monitoring, these findings represent the most sensitive HCN detection reported to date via optical methods, to the best of our knowledge.

## 2 Experiment

**Fig. 1:**
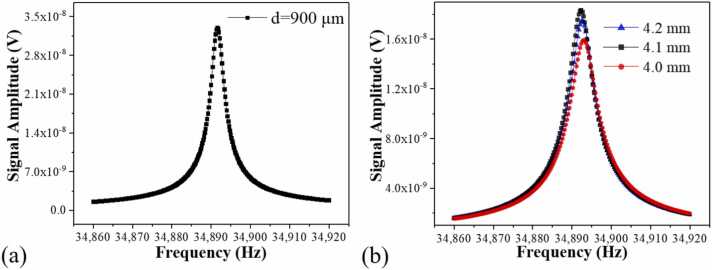
(a) Resonance curve of clamp-type QTF. (b) Resonance curve of clamp-type QTF with the AmRs of varying lengths.

To facilitate easier optical collimation and stronger photoacoustic transducing capability, we adopted a clamp-type QTF as the core of our QEPAS spectrophone. Initial modifications involved the removal of the standard watch QTF's metal casing, resulting in a measured resonance frequency and quality (*Q*) factor of 32,763.2 Hz and 11,584, respectively. A noteworthy adaptation was the inclusion of an aperture of 900 µm in diameter *d*, situated 0.7 mm from the QTF opening. The clamp-type configuration led to a resonance frequency shift to 34,891.6 Hz. However, the *Q* factor was kept at 11,216. The detailed resonance curve for the clamp-type QTF can be seen in Fig. 1(a). Lorentz fitting was used to obtain the resonance peak. The *Q* factor was calculated by dividing the resonance frequency *f*_0_ by its full width at half maximum *Δf*. The resonance was measured at the excitation voltage of 400 mV peak-to-peak.

**Fig. 2:**
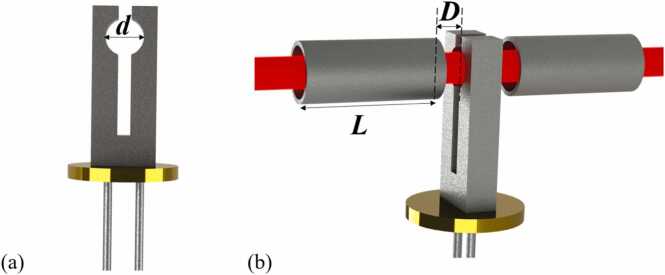
(a) Front view of the clamp-type QEPAS, (b) Schematic diagram of double-tube longitudinal AmRs. *d*: the diameter of the aperture. *L*: the length of AmRs. *D*: the distance between the AmR end and the QTF plane.

To enhance the acoustic signal amplitude with the clamp-type QTF, we chose the on-beam QEPAS configuration with strong photoacoustic coupling, not the off-beam configuration with the advantage of easy optical collimation [53]. The employment of on-beam configuration benefits from the ∼900 μm large aperture of the custom clamp-type QTF, which is 3 times larger than the usually used 3×8 mm standard QTF [51]. A pair of longitudinal AmRs were made by a stainless-steel capillary using an ultra-precision CNC machine tool. The outer diameter and inner diameter of the AmR were 1.2 mm and 1.0 mm, respectively. To ensure the beam passes through the aperture and the resonator clearly, the inner diameter of the resonator was designed to be slightly larger than that of the aperture. To explore the optimal length of the AmR, we made four different AmRs with lengths *L* of 4.0 mm, 4.1 mm, 4.2 mm, and 4.3 mm, respectively. Positioned symmetrically on each side of the clamp-type QTF, the AmRs formed an on-beam configuration in which the laser beam was co-axially directed, with a precise gap *D* = 20 µm maintained from the end of the AmR to the QTF plane, as depicted in Fig. 2(b).

**Fig. 3:**
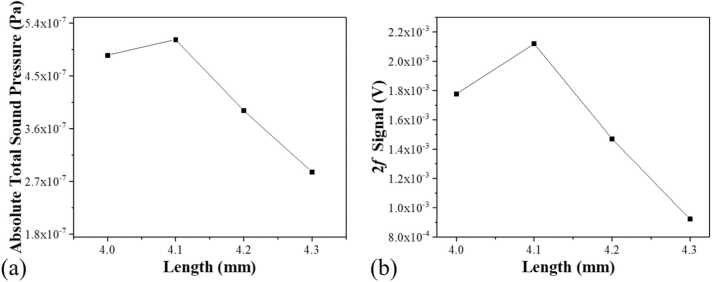
(a) Simulation of the sound pressure curve, (b) 2 *f* signal amplitudes corresponding to the different AmR lengths.

The combined QTF and AmR system constitutes an on-beam QEPAS spectrophone. The resonance curves of the QEPAS spectrophones with different AmR lengths are compared in Fig. 1(b), indicating minimal shifts in resonance frequency. The frequency shift was less than 0.1 Hz. The 4.1 mm AmR demonstrated the highest resonance amplitude and, in combination with photoacoustic detection performance metrics, was determined to be the optimal length. Finite element analysis was conducted using the "Pressure Acoustic, Frequency Domain" module of COMSOL Multiphysics to evaluate the effect of AmR length on sound pressure levels. By varying the dimensions and configurations of the AmR in the simulation, we identified the optimal geometry that maximized sound pressure levels and acoustic coupling efficiency. The QTF was modeled as a cantilever beam, one end of which was fixed, enabling it to vibrate in a flexural mode. By holding the resonance frequency of the clamp-type QTF constant, we measured the absolute total sound pressure intensity produced by varying AmR lengths. The laser excitation was optimally positioned by centering the circular aperture at 0.7 mm below the opening of a standard QTF with dimensions of 3×8 mm. Simulation results, as depicted in Fig. 3(a), indicated the theoretical optimal length of each AmR to be 4.1 mm to maximize absolute total sound pressure. Consequently, the aggregate length of the on-beam longitudinal resonator is 8.2 mm. In the context of a custom clamp-type QTF resonating at 34,891.6 Hz, the optimal total AmR length ranged between half and a full wavelength, accounting for the separation distance from the QTF plane.

**Fig. 4:**
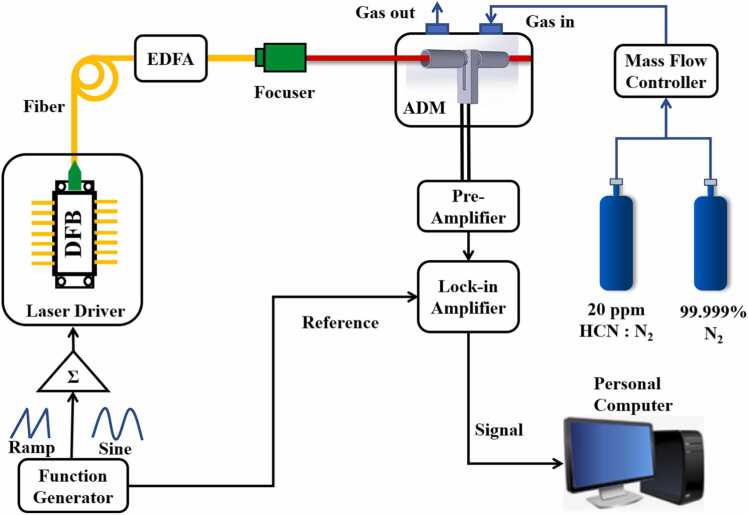
The schematic diagram of the QEPAS system for HCN gas detection.

In this work, a pair of acoustic resonators was coupled with a quartz tuning fork to enhance the signal. The relationship between the *Q* factor of the quartz tuning fork and detection sensitivity is complex. In Fig. 4, the schematic diagram of the *Q* factor measurement is shown. A series of sine signals with constant amplitude but different frequencies were produced by a function generator to drive the tuning fork. The output of the tuning fork was recorded by a preamplifier and a lock-in amplifier. A decrease in the *Q* factor of the tuning fork indicates energy transfer between the tuning fork and the resonator. However, regardless of the presence of a resonator, only the response of the tuning fork was recorded. Thus, the obtained *Q* factor corresponds to the *Q* factor of the tuning fork alone, not the *Q* factor of the coupled tuning fork-resonator system. All the work were carried out at atmospheric pressure.

Verification experiments were conducted using four QEPAS spectrophones with varying AmR lengths to detect H_2_O in ambient air. The air's relative humidity was monitored using an electronic humidity meter. We normalized the photoacoustic signal amplitude against the calculated absolute H_2_O molecule concentration. Fig. 3(b) illustrated the normalized 2 *f* signal amplitudes corresponding to the different AmR lengths. The normalized photoacoustic signal amplitude peaked at an AmR length of 4.1 mm, aligning with our theoretical predictions. Ultimately, we selected a pair of longitudinal AmRs, each with an inner diameter of 1.0 mm and a length of 4.1 mm, to construct the on-beam QEPAS spectrophone. The bare clamp-type QTF without an AmR was measured, and the signal amplitude was found to be 1.56×10^−4^ V. In comparison, with the 4.1 mm AmRs, the signal amplitude increased to 2.12×10^−3^ V. This optimized configuration enhanced the photoacoustic signal amplitude by a factor of 14.6 compared to a bare clamp-type QTF without an AmR. Subsequent studies employed the optimized on-beam QEPAS spectrophone for HCN detection.

The schematic diagram of the QEPAS system for HCN gas detection was shown in Fig. 4. The system utilized a distributed feedback (DFB) diode laser as the excitation source, which emitted at a central wavelength of 1538 nm. This wavelength can be roughly adjusted by temperature changes and finely tuned through injection current. Enclosed in a butterfly package, the laser’s pigtail is fused to the input of an EDFA using an optical fiber fusion splicer. A C-band EDFA, with an amplification capability of 40 dBm, amplifies the optical power emanating from the diode laser. Subsequently, the output from the EDFA was directed through an OZ Optics fiber focuser with an approximate focal length of 1 cm. The aim was to concentrate the amplified laser beam through the QEPAS spectrophone with precision, ensuring that incidental scatter light would not strike the spectrophone, which could impede the system's performance. To enhance the signal-to-noise ratio (SNR) of the detection, a second harmonic (2 *f*) detection technique was implemented [34]. A dual-channel arbitrary waveform generator (Tektronix AFG3102) produced a ramp signal, which, through the Thorlabs CLD1015 laser driver, finely tuned the laser emission wavelength. In parallel, a sine wave at half the resonance frequency (*f*_0_/2) of the QEPAS spectrophone was generated to modulate the laser wavelength, in which *f*_0_ was the resonance frequency of the QEPAS spectrophone. The signals from the clamp-type QTF were first processed by a custom trans-impedance amplifier, which features a feedback resistance of 10 MΩ. To mitigate any electromagnetic interference, the QEPAS spectrophone and the preamplifier were enclosed within a compact, shielded module [65]. The signal was then conveyed to a lock-in amplifier (Stanford SRS 830) and demodulated in the 2 *f* mode. The lock-in amplifier was configured with a time constant of 1 s and a filter slope of 12 dB/octave. Communication with a personal computer (PC) was established via the RS232 serial port for data acquisition and control. For testing, gas mixtures with varying HCN concentrations were prepared using a 99.999 % pure nitrogen (N_2_) gas cylinder and a 20 ppm HCN gas cylinder. The dynamic mixing of gases was performed using a gas dilution system equipped with two mass flow controllers (MFCs). To optimize the signal quality, taking into account noise and gas flow effects within the high-power setup [66], the gas flow rate was maintained at 150 standard cubic centimeters per minute (SCCM). Control over the entire experimental setup was exerted through a custom-developed LabView program.

**Fig. 5:**
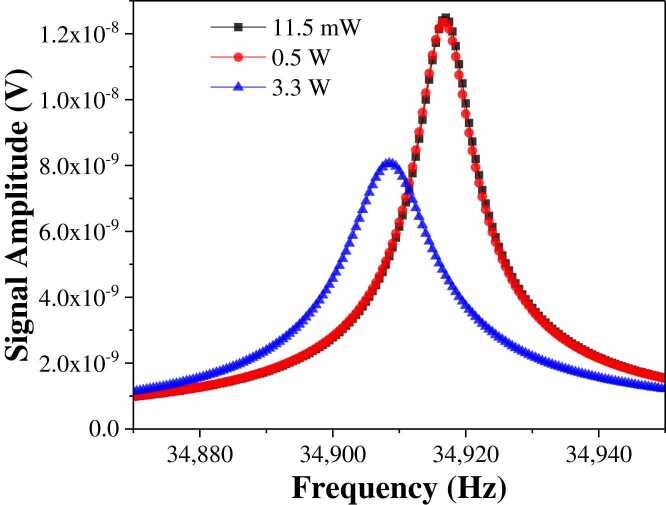
Clamp-type QTF resonance frequency at different laser power.

Before the detection of HCN, it is essential to calibrate the resonance frequency of the QEPAS spectrophone. In this experiment, we evaluated the thermal effects attributed to high excitation power. The diode laser’s output was fixed at 11.5 mW, while the output power from the EDFA was varied from 0.5 W to 3.3 W which is the output power range of the EDFA, from the minimum power output of 0.5 W at this wavelength, to the maximum power the EDFA can amplify. The resonance curves of the spectrophone at different levels of optical excitation power utilizing a consistent electrical drive method were recorded. The results were shown in Fig. 5. The resonance frequency shifts observed at higher laser powers are primarily due to thermal effects induced by increased laser power. However, once the laser power stabilizes, the temperature also reaches a steady state. Therefore, while initial power changes can cause frequency shifts, the system can maintain long-term stability and accuracy as the thermal conditions stabilize. This steady state ensures that the sensor remains reliable over extended periods, with minimal drift in resonance frequency, thus preserving the accuracy of the measurements. With the Lorentz fitting, the resonance frequencies and *Q* factors were summarized in Table 1. As the excitation power increased from 11.5 mW to 0.5 W, the resonance frequency remained relatively stable, at approximately 34,913 Hz. Concurrently, the *Q* factor decreased from 4757 to 4079, with a decline of 14.25 %. However, when the excitation power was further increased from 0.5 W to 3.3 W, there was a remarkable shift in the resonance frequency, descending from 34,913.3 Hz to 34,908.5 Hz. The *Q* factor diminished significantly to 2844, with a 30 % reduction. These findings indicated pronounced thermal effects when the excitation optical power exceeded 3.3 W. Consequently, for the QEPAS system, it is critical to adjust the modulation frequency to account for the thermal effects observed at higher optical excitation powers.

**Table 1:** Parameters of clamp-type QTFs with different laser power.

Laser Power (W)	Frequency (Hz)	*Q* Factor
0.0115	34,913.7	4757
0.5	34,913.3	4079
3.3	34,908.5	2844

**Fig. 6:**
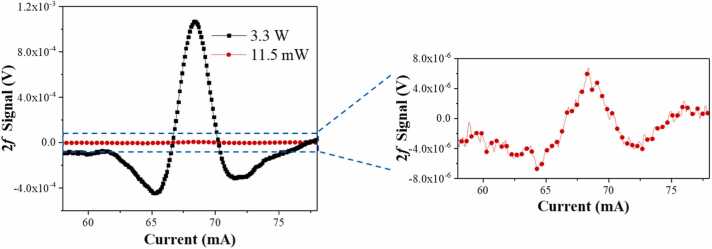
HCN 2 *f* signal of different laser power at a flow rate of 150 SCCM and a concentration of 20 ppm.

Upon reviewing the molecular absorption lines ranging from 6494 cm^−1^ to 6504 cm^−1^ within the HITRAN database [67], an HCN absorption line located at 6501.239 cm^−1^ with an intensity of 5.86×10^−21^ cm/molecule was selected as the target. This target line is devoid of spectral overlap from common atmospheric constituents such as water vapor (H_2_O) and carbon dioxide (CO_2_), ensuring unambiguous detection. A 20 ppm HCN:N_2_ standard gas mixture and a diode laser in conjunction with an EDFA were employed for detection. The diode laser's injection current was tuned between 76 mA and 102 mA by the ramp signal, corresponding to an emission wavelength sweep from 6500.91 cm^−1^ to 6501.82 cm^−1^. The resulting 2 *f* signals were displayed in Fig. 6. Remarkably, the peak signal obtained with a 3.3 W EDFA reached 1.04×10^−3^ V, a substantial enhancement by a factor of 193.47 compared to the 11.5 mW diode laser. Despite a significant increase in optical power, approximately 286-fold, the noise level remained commendably stable. This exemplary noise suppression capability can be ascribed to the custom design of the clamp-type QTF, featuring a 900 μm diameter aperture. This aperture is sufficiently large to allow the laser beam to pass through.

## 3 Results and discussion

**Fig. 7:**
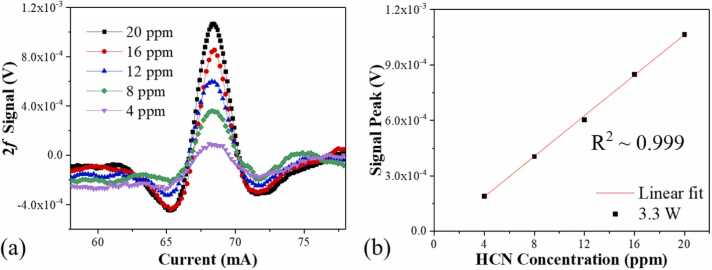
(a) HCN signal amplitude with different gas concentrations, (b) Concentration and signal linearity.

In the subsequent phase, measurements of different HCN:N_2_ gas mixture concentrations were conducted using the EDFA-boosted QEPAS sensor to validate its performance. Concentrations ranging from 4 ppm to 20 ppm of HCN gas were prepared to utilize the aforementioned gas dilution system. The power of the EDFA was set as 3.3 W, and the laser modulation depth was set as 2.28 cm^−1^. In our experiments, we have tested HCN concentrations up to 20 ppm, and the sensor demonstrated a linear response throughout this range. Therefore, the upper limit of HCN concentration that the sensor can accurately detect is up to 20 ppm, ensuring reliable performance across both low and relatively high concentration levels. The obtained results were displayed in Fig. 7(a), where an increase in HCN concentration was observed to proportionally enhance the 2 *f* signal. The experiments were replicated three times, and from these, the 2 *f* signal peaks were extracted from the waveforms. These peaks were then used to construct the response curve, as illustrated in Fig. 7(b). A linear fit was applied to the gas concentration-response data, from which an R-square value of 0.999 was derived, indicating the sensor’s robust linearity.

**Fig. 8:**
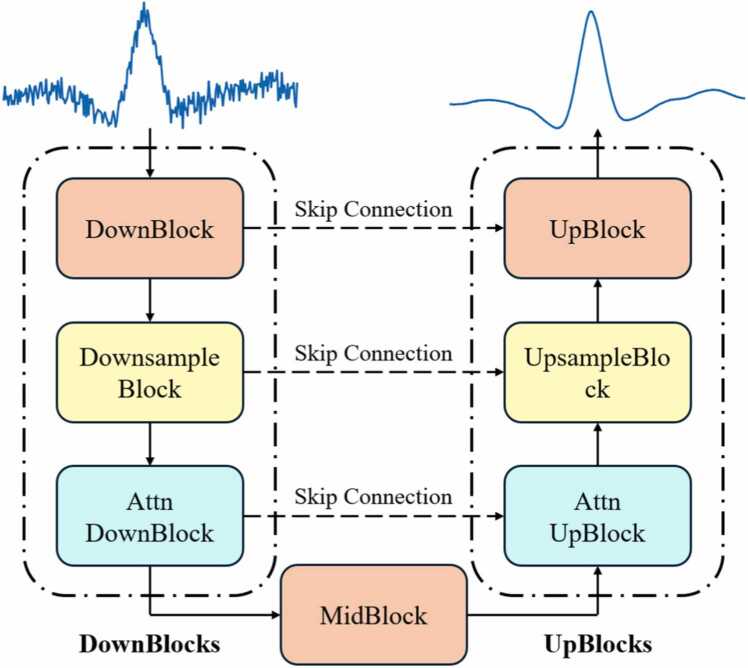
The complete architecture of U-net.

The implementation of a neural network filtering method based on deep learning, known as the U-net, was undertaken to enhance the performance of the QEPAS sensor. This innovative machine-learning method dynamically assigns varying weights to individual sampling points, aiming to streamline the complex manual parameter evaluation process and enhance the effectiveness of spectral filtering [68]. The U-net neural network offers significant advantages over traditional noise reduction methods. Traditional methods, such as Fourier or wavelet transforms, often require manual tuning of parameters and may not effectively handle complex noise patterns. In contrast, the U-net neural network automatically learns the noise characteristics from the data, providing more accurate and adaptive noise reduction. While the U-net approach has higher computational complexity due to the deep learning model, it is more efficient in terms of noise suppression and preserving signal integrity. The U-net model's ability to perform end-to-end learning and its robustness in dealing with various noise types make it a superior choice for our application, ensuring high sensitivity and low detection limits. As shown in Fig. 8, the 2 *f* signals will first undergo dimensional reduction upon its import into the training set. This process involves data sampling and feature extraction, facilitated by skip connections. Initially, the data are passed through the dimensioning component to prepare for subsequent data combinations. This step is followed by repeated dimensionality reduction until reaching the lowest dimensionality. Subsequently, the data undergoes dimensional uplift to return to its original dimensionality. The results of this uplift are then combined with the results of the initial reduction to obtain optimized outcomes. This iterative process of dimensionality uplift continues until the data achieves its final optimized state. The U-net was implemented using the Pytorch deep learning framework. U-net consists of DownBlocks, MidBlocks, and UpBlocks. DownBlocks is responsible for compressing the dimension of the input one-dimensional signal from 1 × 1 × 256–1 × 64 × 64 to achieve the role of information extraction. MidBlock is a bottleneck layer. This layer is responsible for reducing the dimensionality of the input data, compressing the information, and extracting features. This compression helps in reducing the computational complexity and memory requirements of the network, making it more efficient. UpBlocks is responsible for outputting the denoised 2 *f* signal through the hidden state from DownBlocks and MidBlock. The detailed information of the U-net is presented in Table 2. During the algorithm training process, we used a batch size of 256, an Adam optimizer with a learning rate of 0.0001, and performed 39, 000 steps (shown in Table 3). All training and testing were conducted on an NVIDIA 2080 Ti GPU. The data source for training the U-net model comprised experimentally obtained photoacoustic signals of varying hydrogen cyanide (HCN) concentrations under different environmental conditions. The parameters of the U-net model were obtained from the Unet1DModel in diffusers (https://huggingface.co).

**Table 2:** The detailed information of the U-net.

Input – 1 × 1 × 256		
DownBlocks	DownBlock	Resnets – 1 × 32 × 256
	DownsampleBlock	Downsample – 1 × 32 × 128
		Resnets – 1 × 32 × 128
	AttnDownBlock	Downsample – 1 × 32 × 64
		Resnets & attentions – 1 × 64 × 64
MidBlock	Downsample – 1 × 64 × 32	
	Resnets & attentions – 1 × 64 × 32	
	Upsample – 1 × 64 × 64	
UpBlocks	AttnUpBlock	Resnets & attentions – 1 × 32 × 64
		Upsample – 1 × 32 × 128
	UpsampleBlock	Resnets – 1 × 32 × 128
		Upsample – 1 × 32 × 256
	UpBlock	Resnets – 1 × 1 × 256
Output – 1 × 1 × 256

**Table 3:** U-net training parameters.

Batch Size	Steps	Optimizer	Learning Rate
256	39,000	Adam	0.0001

**Fig. 9:**
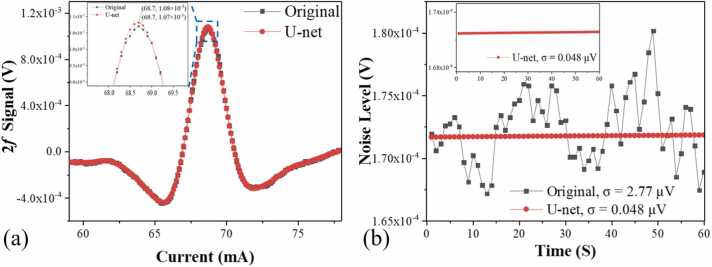
(a) 2 *f* signal amplitude with and without U-net, inset: the zoom of the peak 2 *f* signal amplitude (b) Noise levels with and without U-net, inset: the zoom of the noise obtained with U-net noise filtering.

Unlike conventional machine learning methods based on U-shaped networks, which analyze spectral data at a global level, enabling them to consider dependencies and relationships across all data points, this approach enhanced the algorithm’s ability to discern critical features within spectra. As a result, it achieved more accurate and reliable filtering of HCN absorption information, even in the presence of noise [69]. By first reducing the data to a low latitude and then elevating it to a high latitude, a connection was established between the descending and ascending dimensions. Utilizing this connection, U-net noise filtering was implemented without decreasing the peak value of the signal, as illustrated by the U-net filtered noise graph in Fig. 9. Fig. 9(a) illustrates the peak comparison between the original 2 f signal and the U-net processed 2 *f* signal. The inset of Fig. 9(a) is the zoom of the 2 *f* signal peak. From the inset, it can be observed that the peak of the original signal is 1.08×10⁻³ V, whereas the peak of the U-net processed signal is 1.07×10⁻³ V, showing a difference of less than 1 %. The obtained noise was shown in the Fig. 9(b). In regions far from the molecular absorption lines, the sensor noise is relatively low due to the lack of absorption. In this experiment, to simulate real-world continuous monitoring scenarios and obtain the true noise of the sensor, the laser wavelength was locked at the molecular absorption line and filled the photoacoustic cell with pure nitrogen to evaluate the noise level. It was observed that the signal level remained nearly constant, while a significant reduction in the 1*σ* noise level was noted, from 2.77 μV to 0.048 μV. The detection SNR and the Normalized Noise Equivalent Absorption (NNEA) coefficient were tabulated in Table 4. The results indicated that the detection SNR could be augmented by a factor of 58. Conclusively, with a 1-second integration time, the sensor achieved a 1*σ* detection limit and an NNEA coefficient of 0.89 ppb and 2.31×10^−9^ W·cm^−1^·Hz^−1/2^, respectively. The baseline noise level of our QEPAS system is 0.048 μV, which significantly influences the detection limit. This low noise level, achieved through the optimized design of the quartz tuning fork and the implementation of the U-net noise filtering technique, allows for the detection of weaker signals and enhathences the system's sensitivity. The low noise floor is essential for accurately detecting trace concentrations of hydrogen cyanide (HCN), ensuring high sensitivity and reliability in our measurements. Compared with reference [14], which reports an MDL of 29 ppb, our results may initially appear to have a gap. However, it is important to note that our experiments used different equipment. The application of the U-net noise filtering technique significantly improved our detection sensitivity. Although there is a difference, 29 ppb and 51.77 ppb are within the same order of magnitude, demonstrating that our approach remains competitive.

**Table 4:** Comparison table original signal and U-net signal. DL: detection limit.

	Signal (V)	1σ Noise (V)	SNR	DL (Ppb)	NNEA (W·cm^−1^·Hz^−1/2^)
Original	1.07×10^−3^	2.77×10^−6^	386.28	51.77	1.32×10^−7^
U-net	1.08×10^−3^	4.82×10^−8^	22406.63	0.89	2.31×10^−9^

Table 5 provides a comparative analysis of the performance of various mainstream. HCN sensors developed in recent years, with a particular emphasis on response time and detection sensitivity. Optical detection methods, represented in the table, are notable for their relatively rapid response, typically on the order of seconds, as well as their impressive detection thresholds. Optical sensors employed mid-infrared optical parametric oscillator or integrated cavity can achieve a sensitivity of ppb level. Traditional QEPAS sensor obtained a detection sensitivity of 21 ppb with 1 s integration time [14].

**Table 5:** Comparison of various HCN gas sensors.

Method	Average time	Sensitivity	Ref.
Ion mobility spectrometer	A few seconds	20 ppb	[70]
Polymer nanocomposite material	21 seconds	890 ppb	[71]
Resonance silicon film	\	2 ppm	[72]
Quartz crystal microbalance sensor	1 second	169 ppb	[73]
Colorimetric paper	5 minutes	10 ppb	[74]
Fluorometric detection	10 seconds	60 ppm	[75]
Photonic crystal cavity	\	2 ppm	[76]
Tunable diode laser absorption spectroscopy	\	1 ppm	[77]
Traditional QEPAS	1 second	21 ppb	[14]
Mid-infrared optical parametric oscillator-based photoacoustic spectroscopy	1 second	0.19 ppb	[78]
Off-aixs integrated cavity output spectroscopy	1 second	8 ppb	[79]
Clamp-type QEPAS	1 second	0.89 ppb	This work

In this work, a lower-cost clamp-type QTF with optimized dual tube AmRs were developed. The sensor handles potential interference from other gases through several key mechanisms. First, the QEPAS system is highly selective due to the precise tuning of the laser wavelength to the specific absorption line of hydrogen cyanide (HCN). This minimizes the likelihood of interference from other gases. Additionally, the high *Q*-factor of the quartz tuning fork (QTF) enhances the system's selectivity by narrowing the detection bandwidth. Furthermore, the use of advanced signal processing techniques, including the U-net neural network, helps to further distinguish the HCN signal from background noise and potential interfering signals. These combined features ensure that the sensor can accurately detect HCN even in the presence of other gases commonly found in industrial or environmental settings. The rapid response time of 1 second for HCN detection in our study was measured using the lock-in amplifier setup. The lock-in amplifier was configured with a time constant of 1 second, which directly determines the system's response time by defining how quickly the output signal stabilizes in response to changes in the detected gas concentration [80]. Factors contributing to achieving this rapid response include the high sensitivity and fast reaction of the quartz tuning fork (QTF), the efficient acoustic coupling in the QEPAS setup, and the advanced signal processing capabilities of the U-net neural network for noise filtering. These elements combined ensure that the sensor can promptly and accurately detect changes in HCN concentration within 1 second. Moreover, when compared to conventional HCN sensors such as Ion mobility spectrometer, the compact form factor of the QEPAS sensor described herein is highlighted, offering significant advantages for the development of portable and miniaturized gas sensors. The compact size is achieved through the use of a clamp-type QTF and a dual-tube AmR, both of which are small in size. The high *Q*-factor of the QTF enhances sensitivity without requiring bulky components. Additionally, the sensor is designed for easy integration into handheld devices, facilitating real-time, on-site gas detection and monitoring. The low power consumption, supported by efficient acoustic coupling and advanced noise filtering techniques like the U-net neural network, further underscores its suitability for portable applications.

## 4 Conclusions

In conclusion, this paper introduced a novel clamp-type QTF with an on-beam AmR for QEPAS and demonstrated its superior performance in detecting hydrogen cyanide (HCN) gas. The innovative QEPAS sensor, utilizing the clamp-type QTF, exhibited enhanced sensitivity and a rapid response. Optimized through both theoretical and experimental approaches, the sensor achieved a substantial increase in photoacoustic signal amplitude, with an optimized AmR length of 4.1 mm. The inclusion of an EDFA and a neural network filtering method based on machine learning, known as the U-net significantly improved the detection SNR, achieving a minimum detection limit of 0.89 ppb and an NNEA coefficient of 2.31×10^−9^ W·cm^−1^·Hz^−1/2^. This performance enhancement was realized without saturation effects under a 3.3 W excitation laser, suggesting potential for further advancements with higher power outputs. The sensor’s efficiency, coupled with the cost-effectiveness of using commercial watch QTF, positions the clamp-type QTF as a viable solution for real-time HCN monitoring and indicates its potential for miniaturization in portable devices. The proposed QEPAS sensor system offers significant potential for miniaturization and portability due to its compact form factor, high *Q*-factor, and advanced noise filtering techniques, which collectively ensure high sensitivity, low power consumption, and easy integration into handheld devices for real-time, on-site gas detection and monitoring. This work not only contributes to the advancement of gas sensing technology but also opens avenues for broader applications in industrial safety and environmental monitoring. Further improvement can be made by using of novel light sources such as dual comb sources or high-power single-longitudinal-mode solid-state lasers [81], [82].

## CRediT authorship contribution statement

**Jianhui Yu:** Conceptualization. **Lihao Wang:** Writing – review & editing, Writing – original draft, Data curation, Conceptualization. **Yongchun Zhong:** Conceptualization. **Bin Liu:** Conceptualization. **Yaohong Zhao:** Conceptualization. **Chenglong Wang:** Conceptualization. **Huadan Zheng:** Writing – review & editing, Conceptualization. **Haohua Lv:** Data curation. **Jiabao Xie:** Conceptualization. **Wenguo Zhu:** Conceptualization. **Huijian Luo:** Conceptualization. **Haoyang Lin:** Conceptualization.

## Declaration of Competing Interest

We wish to confirm that there are no known conflicts of interest associated with this publication and there has been no significant financial support for this work that could have influenced its outcome.

## Data Availability

Data will be made available on request.
